# Distribution of multi-level B cell subsets in thymoma and thymoma-associated myasthenia gravis

**DOI:** 10.1038/s41598-024-53250-6

**Published:** 2024-02-01

**Authors:** Peng Zhang, Yuxin Liu, Si Chen, Xinyu Zhang, Yuanguo Wang, Hui Zhang, Jian Li, Zhaoyu Yang, Kai Xiong, Shuning Duan, Zeyang Zhang, Yan Wang, Ping Wang

**Affiliations:** 1 Department of Cardiovascular Thoracic Surgery, Tianjin Medical University General Hospital, Anshan Road No. 154, Heping District, Tianjin, 300052 China; 2 School of Medicine, University of Dundee, Dundee, UK; 3 Tianjin Ruichuang Biological Technology Co. Ltd, Tianjin, China

**Keywords:** Surgical oncology, Autoimmune diseases

## Abstract

B-cell subsets in peripheral blood (PB) and tumor microenvironment (TME) were evaluated to determine myasthenia gravis (MG) severity in patients with thymoma-associated MG (TMG) and the distribution of B cells in type B TMG. The distribution of mature B cells, including Bm1–Bm5, CD19^+^ and CD20^+^ B cells and non-switched (NSMBCs) and switched (SMBCs) memory B cells, were determined in 79 patients with thymoma or TMG. Quantitative relationships between the T and TMG groups and the TMG-low and TMG-high subgroups were determined. NSMBCs and SMBCs were compared in TME and PB. Type B thymoma was more likely to develop into MG, with types B2 and B3 being especially associated with MG worsening. The percentage of CD19^+^ B cells in PB gradually increased, whereas the percentage of CD20^+^ B cells and the CD19/CD20 ratio were not altered. The (Bm2 + Bm2′)/(eBm5 + Bm5) index was significantly higher in the TMG-high than in thymoma group. The difference between SMBC/CD19^+^ and NSMBC/CD19^+^ B cell ratios was significantly lower in the thymoma than TMG group. NSMBCs assembled around tertiary lymphoid tissue in thymomas of patients with TMG. Few NSMBCs were observed in patients with thymoma alone, with these cells being diffusely distributed. MG severity in patients with TMG can be determined by measuring CD19^+^ B cells and Bm1-Bm5 in PB. The CD19/CD20 ratio is a marker of disease severity in TMG patients. Differences between NSMBCs and SMBCs in PB and TME of thymomas can synergistically determine MG severity in patients with TMG.

## Introduction

Thymoma, an epithelial cell tumor of the thymus, is one of the most common anterior mediastinal tumors in adults and is often combined with myasthenia gravis. Patients with thymoma-associated MG (TMG) typically have more severe symptoms and a higher frequency of systemic disease with medullary and respiratory symptoms^1^. Early resection of thymoma is necessary to relieve MG symptoms and can significantly improve the prognosis of patients with thymoma^2^. However, MG may persist after thymoma removal. Thymus-associated B-cell clones and plasma cells persist in the circulation after thymectomy, and their persistence may explain the incomplete response to resection^3^.

Two separate subsets of B cells are present in the human thymus, located in the medullary and perivascular spaces (PVS). PVS B cells mainly consist of class-switched IgG^+^CD27^+^ memory cells, the main subset of thymic B cells in adults. Mutational analyses of B cell subsets delineated by IgM and IgD in peripheral blood (PB) have shown that these PB B cells are composed of nonmutated naive B cells and somatically mutated memory B cells. CD27 was shown to be a useful marker for PB B cells that have undergone somatic hypermutation and is therefore regarded as a new marker for memory B cells (Bmems)^4^.

CD19 is expressed throughout B cell development. Mature B lymphocytes have been classified as Bm1 to Bm5, depending on the comparative membrane expression of IgD and CD38 during B lymphocyte maturation^5^. Initially, naive B cells (NBCs) develop in the spleen. Following antigen (Ag) activation, these NBCs enter the incipient germinal centers (GCs). Finally, Bmems or plasma cells with highest Ag-affinity emerge from the GCs. In addition, some NBCs could be activated in extrafollicular areas, in particular the T cell area or medullary cords, rapidly differentiating into plasma cells^6,7^. Human peripheral memory B cells can be grouped according to their expression of CD27 and IgD into CD27^+^IgD^+^ pre-switched, CD27^+^IgD^-^ post-switched and CD27-IgD- double negative memory B cells^7^. Although CD19^+^CD20^−^ cells mainly develop into memory cells in lymphoid tissues, these cells were found to be present in primary and secondary lymphoid tissues, allowing their entrance into the circulation. GCs are regarded as the major sites for the development of autoimmune B cells. Targeting extrafollicular responses may play a vital role in treating B cell-mediated autoimmune diseases^8^. GC constitutes a site of antibody affinity maturation through processes of clonal proliferation, somatic mutation and selection and is largely observed in secondary lymphoid organs. GC is sometimes observed in inflammatory organs called tertiary lymphoid organs, and it has been shown that this tissue is also observed in the thymus of patients with AChR + early-onset myasthenia gravis^9^.

Tertiary lymphoid tissue (TLT), also called ectopic lymphoid tissue, forms at the site at which immune responses are exacerbated in autoimmune diseases, chronic inflammation and several tumor types^10,11^. The tumor microenvironment (TME), which consists of tumor‐infiltrating immune cells (TIIC), tumor cells, and stromal cells, is a complex immune network^12^. In particular, CD20^+^ B cells have been found in the TLTs of tumors^13^. The frequency of Bmems was shown to be significantly higher in tumoral immune-response regions, whereas NBCs were located in non-responders. TLTs were found to contain high numbers of proliferating B cells, indicating the formation of active GCs^13,14^. Although many tumor-infiltrating B cells have been identified, their specific functions and their relationships with tumors are unclear^15–17^. According to one hypothesis, tumor-infiltrating B cells promote tumorigenesis, whereas, according to another hypothesis., these cells may correlate positively correlated with improved prognosis in tumor patients and are associated with TLTs^14,18^.

The present study compared the differential distribution of B cell subsets (including Bm1-Bm5, non-switched memory and switched memory B cells in PB) in patients with thymoma alone and those with TMG, as well as in the TMG-low (TMGL) and TMG-high (TMGH) subgroup. This study identified new indicators that are important to evaluate the severity of MG in patients with TMG patients. The numbers of switched and non-switched memory B cells were compared in PB and tissues. These findings suggested that TMG was characterized by the recruitment non-switched memory B cells in peripheral blood to the TME of the thymus. These cells subsequently participated in the formation of TLTs and differentiated into memory B cells and plasma cells through the growth of GCs, suggesting that their quantity and distribution are closely related to the severity of MG in thymoma.

## Materials and methods

### Patients

A total of 93 patients with thymoma between February 2019 and March 2021 were included in the study. Fourteen of these patients were excluded, four because their blood samples were wrong, two because, sufficient clinical details were unavailable, and eight because postoperative pathology showed thymic carcinoma. Of the 79 remaining patients, 38 had thymoma alone and 41 had TMG. A sample was retained for B-cell phenotyping as soon as examined for serological tests, associated with conventional examination for myasthenia gravis in the Cardiovascular Thoracic Surgery Department at Tianjin Medical University General Hospital. All the specimen was acquired following the declaration of Helsinki and adherence to guidelines from the local ethical committee (IRB2019-KY-179). A double-blind course was formulated to analyze the subsets of B-cell distribution. B-cell subsets were surveyed by the laboratory staff without the awareness of the patients’ diagnosis, which was concurrently confirmed by the clinical doctor without the awareness of the subsets of B-cell distribution. Division of the thymoma associated with MG patients into two groups (TMGL (Osserman type I and IIa), TMGH (Osserman type IIb, III and IV)) according to the clinical evaluation of the patients’ extent of myasthenia gravis. Inclusion criteria: (1) age ≥ 18 years old; (2) CT examination confirmed anterior mediastinal mass; (3) pathologically confirmed thymoma. Exclusion criteria: (1) age < 18 years old; (2) comatose patients/patients with cognitive impairment; (3) severe malnutrition; (4) with other severe heart, lung, liver, kidney disease Insufficiency; (5) with other malignant tumors.

### Flow cytometry analysis

PBMCs were isolated by Ficoll density gradient centrifugation according to the manufacturer’s instructions (Hao Yang Biological Manufacture Co., Ltd., Tianjin, P.R. Chian). Incubate with anticoagulant antibody at room temperature for 20 min, lysate the erythrocytes with hemolysin, and then wash and resuspend them with PBS and then test them on the machine. The specificity of the antibodies and compensation were confirmed by the isotype control. Stained cells were analyzed by FCM (FACSCanto II; BD Biosciences, San Jose, CA, USA) after removing dead cells. Data analysis was performed using Cell Quest software (BD Biosciences).

### Immunohistochemical staining

#### Multi-label immunofluorescence

Thymoma tissue was paraffin-embedded and sectioned. Multiplex staining of thymoma tissue was completed using PANO 4-plex IHC kit (Cat# 10001100100, Panovue) according to the manufacturer’s instruction. The paraffin sections were deparaffinized, dehydrated, antigenically repaired and then closed with 5% BSA, after which the sections were incubated with a primary antibody at 4 °C for one night, then taken out and rewarmed at room temperature, incubated with HRP secondary antibody, washed and then stained by adding the dye of the corresponding panel, and so on, and so forth for several rounds of index staining, and then finally the nuclei of the cells were ransed by using DAPI, and then finally the slices were blocked. CD19 was used to tag the whole B cells. Non-switched B cells were performed by CD19, CD27, and IgD positive expression. Meanwhile, switched memory B cells were CD19 and IgD- positive expressions, which were CD27- negative expressions.

### Digital image acquisition and analysis

The stained sections were digitally scanned at an absolute magnification of × 40 using the Mantra System (PerkinElmer). The latter captures the fluorescent spectra at 20 nm wavelength intervals from 420 to 720 nm with duplicate exposure time. At least 5 fields of immunocyte enriched tumoral area were chosen for image capture and were analysed with inform 2.4.4 (Akoya Biosciences). Multispectral images were split by the spectral libraries built from images of single-stained slides.

### Statistical analysis

Data were checked for normality with the Kolmogorov–Smirnov or Shapiro–Wilk test, the mean difference between the two groups were tested using 2-dependent sample Student’s t-test if the variables were normally distributed. One-way analysis of variance (ANOVA) test was used for the statistical analysis of more than two groups’ differentiations. For continuous variables, normally distributed data were represented by mean and standard deviation (Mean ± SD). For data that were not normally distributed, Median and interquartile range (Q3–Q1) will be applied to describe the data (Q3–Q1).

For continuous data that did not follow normal distribution, the Median Test or the Independent-Samples Kruskal–Wallis Test was conducted. Mann–Whitney U test was also used for comparing two groups of continuous data that were not normally distributed. Cross-tabulations were used to analyze the statistical correlation between categorical variables. Significance was considered at an alpha value of 0.05. All statistical analyses were performed by SPSS V26.0. The Bonferroni adjustment, which adjusted *P* value by times of tests (n), was used accounting for multiple testing. Adjusted *P* value equalled to 0.05/n. The alpha level was set at 0.05/n to determine two-tailed significance. The exact value of *P* was shown in the figure legends. The representations of *P* were shown in the figure legends. The Prism software (GraphPad Software) version 8 was used for data analysis and graphing data.

### Ethical approval

The experimental protocol was established, according to the ethical guidelines of the Helsinki Declaration and was approved by the Ethics Committee of Tianjin Medical University General Hospital. The approval number was IRB2019-KY-179 (Apr. 25th, 2019).

### Informed consent

Informed consents were obtained from all patients for the use of their samples.

## Results

### Pathological B-cells were present mostly in thymomas associated with myasthenia gravis

#### Baseline

This study included 93 patients with an ESMO (European society for medical oncology)-based diagnosis of thymoma^19^, including 46 with and 47 without MG^20^. These 79 patients included 45 (57.0%) women and 34 (43.0%) men (Fig. S1); their baseline characteristics are illustrated in Table S1.

Because cell populations were measure in both PB and tissue samples, comparisons of P values were subjected to Bonferroni correction. Cell populations were tested 32 times (n_PB_) in PB, with adjusted P values equal to 0.05/32 (0.002). Cell populations were tested four times (n_TME_) in tissue samples, with adjusted *P* values equal to 0.05/4 (0.013).

Masaoka stage, tumor volume and tumor texture of the thymomas did not differ significantly in patients with thymoma alone and TMG (Table S2). MGFA score analysis of all patients with MG showed that patients with more severe MG were more likely to have grade IIIa (Table S2).

Evaluation of the autoimmune status of all patients showed no significant differences among the thymoma, TMGH and TMGL groups in PB concentrations of IgG, IgA, IgM, IgE, C3, C4, CRP, ALB, α1-globulin, α1-globulin, α2-globulin, β1-globulin, β2-globulin, γ-globulin, A/G and ANA antibodies. Most patients positive for AChR antibodies before thymectomy were concentrated in the TMGL and TMGH groups, whereas most patients with thymoma alone were negative for AChR antibodies, a difference that was statistically significant (Table S3).

#### Differences in pathological type

The patients were divided into three groups: those with thymoma (T) alone, thymoma associated with less severe MG (TMGL), and thymoma associated with more severe MG (TMGH), as determine by two clinicians. The pathologic AB type thymoma was most frequent in the T group, whereas the pathologic B type was the most frequent in the TMGL and TMGH groups. Worsening of MG was associated with increased prevalence of B type thymoma, especially of the B2 and B3 types. Severe MG was not always combined with AB or B1 type thymoma, which was replaced by B3 type. Severe MG was more likely to be combined with a mixed pathologic type of thymoma, especially B2B3 type. The difference was statistically significant (Table S2).

### B cells in peripheral blood—CD19 and CD20

Flow cytometry showed that CD19^+^ and CD20^+^ B cells were present in the PB of all 79 enrolled patients. The percentage of CD19^+^ B cells in lymphocytes was slightly higher in PB samples from the TMG than the T group, although the difference was not statistically significant (9.54 ± 3.47 vs. 8.07 ± 4.41, *P* = 0.116) (Table S4). In addition, the ratio of CD20^+^ B cells to lymphocytes in PB did not differ significantly.

The effects of MG severity on B cell populations in PB were evaluated by comparing the ratios of CD19^+^ and CD20^+^ B cells to lymphocytes in the three patient groups (T, TMGL, TMGH). The CD19+/lymphocyte ratio was higher in the TMGH than in the T and TMGL groups, whereas the CD20+/lymphocyte ratios did not differ significantly in these three groups. These findings suggested that the numbers of antibody-secreting CD19^+^ and CD20+ cells, consisting of plasmablasts and plasma cells, respectively, in PB differ are dependent on the severity of TMG (Table 1).

**Table 1:** The expression levels of CD19 and CD20 in peripheral blood.

	T	TMGL	TMGH	Statistical test	Δmean (95%CI of Δmean) (T VS TMGL)	Δmean (95%CI of Δmean) (T VS TMGH)	Δmean (95%CI of Δmean) (TMGL VS TMGH)	*P* value
CD19	8.1 ± 4.4	8.6 ± 3.9	10.5 ± 2.7	F = 2.451, df = 2	− 0.544 (− 2.734, 1.645)	− 2.444 (− 4.669, − 0.218)	− 1.899 (− 4.389, 0.590)	0.094
CD20	7.9 ± 4.3	8.7 ± 3.1	8.4 ± 3.4	F = 0.359, df = 2	− 0.381 (− 3.183, 2.421)	− 1.172 (− 3.930, 1.586)	− 7.914 (− 3.960, 2.378)	0.700
CD19/CD20	1.1 (1.4–0.9)	1.0 (1.3–0.9)	1.2 (1.7–1.1)	H = 3.877^a^ df = 2	–	–	–	0.144

^a^The data did not follow a normal distribution and the Kruskal–Wallis test was used. The statistic of Kruskal–Wallis H Test was 3.877. In peripheral blood, the Bonferroni corrected *P* value was 0.05/32 (0.002).

The gap between CD19^+^ and CD20^+^ cells was amplified by calculating the CD19+/CD20+ ratios in PB. The proportion of CD19^+^ B cells in PB gradually increased, and the CD19+/CD20+ ratio tended to decrease and then increase in thymoma with the aggravation of MG. Moreover, this ratio was higher in the TMGH than in the TMGL group. This difference may be related to the increase in antibody-secreting cells in PB and the migration of early B cells to target organs in the TMGH group (Table 1).

These results suggested that CD19+ and CD20+ populations and CD19+/CD20+ ratios may differ in patients with different pathological types. However, none of these parameters differed significantly among different pathological types (Table S4). In addition, MG severity did not interact with pathological type for CD19+ (F_CD19_ = 1.140, *P*_CD19_ = 0.350) and CD20+ (F_CD20_ = 0.991, *P*_CD19_ = 0.452) populations and CD19+/CD20+ ratios (F_CD19/CD20_ = 0.372, *P*_CD19/CD20_ = 0.931) (Table S5). Main effects analysis indicated that MG severity had a statistically significant effect only on CD19 (F = 4.257, *P* = 0.018). Further pairwise comparisons found that there was a significant difference between the T and TMGH groups (*P* = 0.001), but not between any other pairs of groups (Table S6).

### Bm1–Bm5 in peripheral blood

#### The effect of MG on Bm1–Bm5 in peripheral blood

**Figure 1:**
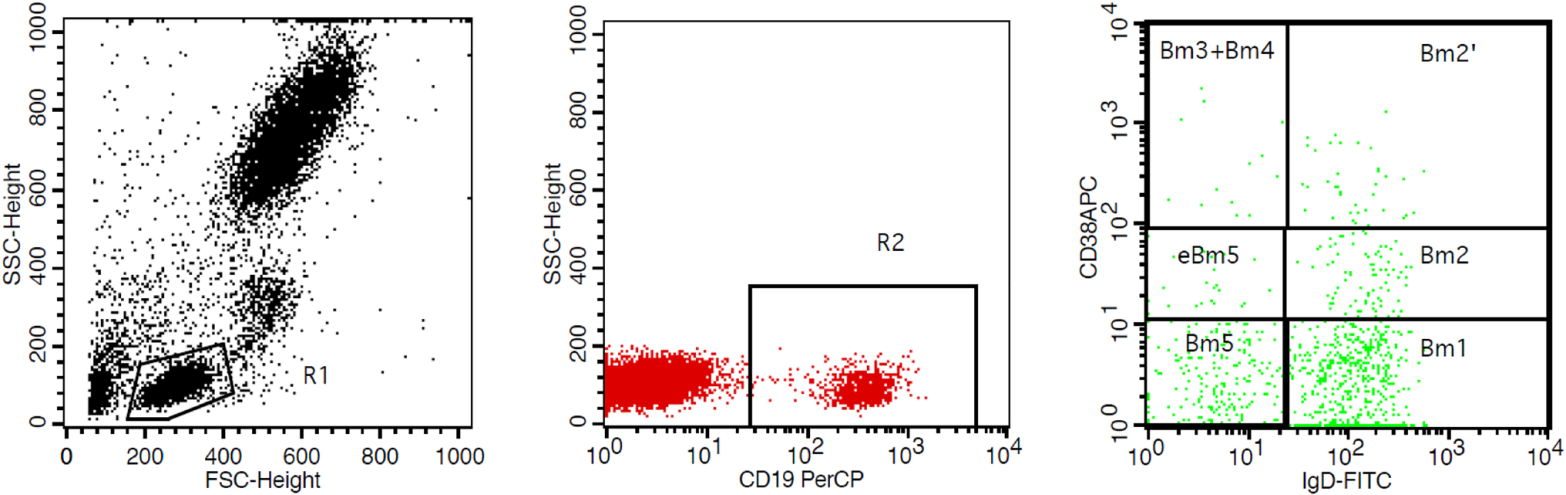
Detection of Bm1–Bm5 in peripheral blood by flow cytometry. Schematic diagram of Bm1–5 flow cytometry analysis results. Expression of IgD and CD38 distributes mature B cells into sequential subsets from Bm1 through Bm5. All the B cell subsets were from CD19^+^-gate. CD19^+^ B cells (R2) were selected from the lymphocyte gate (R1). The positions of the gates are boxed against blank isotype controls for the corresponding antibodies. The B cell lineage subsets were CD19^+^CD38^−^IgD^+^Bm1, CD19^+^CD38^low^IgD^+^Bm2, CD19^+^CD38^hi^IgD^+^Bm2′, CD19^+^CD38^hi^IgD^-^Bm3 + Bm4, CD19^+^CD38^low^IgD^-^eBm5, CD19^+^CD38^-^IgD^−^Bm5.

B-cell subsets were evaluated in 79 patients to determine the relationships between MG severity and the distribution of mature B cells in PB. Based on the expression of CD38, B cell subsets were divided into three groups: CD38^−^, CD38^low^, and CD38^high^. Mature B lymphocytes were classified as Bm1 through Bm5 according to the relative membrane expression of IgD and CD38^21^ (Fig. 1).

To investigate the influence of MG, patients were divided into two groups, the T and TMG groups. The Bm2 and Bm2 + Bm2 subsets were higher, and the Bm3 + Bm4, eBm5, Bm5, and eBm5 + Bm5 subsets were lower, in the TMG than in the T group. In addition, the (Bm2 + Bm2)/eBm5 + Bm5) ratio differed significantly in these two groups (Table 2).

**Table 2:** The expression levels of B cell subsets in peripheral blood.

Subsets of B Cells	T	TMG	*t-value*	*df*	Δmean	95%CI of Δmean	*P* value
Bm1	10.0 ± 6.2	10.7 ± 11.4	− 0.257	56	− 0.694	(− 6.100, 4.711)	0.798
Bm2	3.3 ± 1.5	4.9 ± 2.5	− 2.626	57	− 1.540	(− 2.715, − 0.366)	0.011
Bm2′	2.2 ± 0.4	2.3 ± 0.8	− 0.903	56	− 0.157	(− 0.506, 0.191)	0.328
Bm2 + Bm2′	5.2 ± 1.7	6.9 ± 3.1	− 2.224	53	− 1.665	(− 2.963, − 0.367)	0.013
Bm3 + Bm4	21.0 ± 3.9	17.8 ± 3.7	3.132	56	3.159	(1.138, 5.179)	0.003
eBm5	9.0 ± 2.8	7.7 ± 2.7	1.777	56	1.306	(− 0.167, 2.779)	0.081
Bm5	9.6 ± 2.6	8.3 ± 2.5	1.900	59	1.270	(− 0.067, 2.607)	0.062
eBm5 + Bm5	17.9 ± 4.6	16.2 ± 4.0	1.661	63	1.777	(− 0.362, 3.916)	0.102
Ratio (Bm2 + Bm2′)/(eBm5 + Bm5)	0.3 ± 0.1	0.4 ± 0.2	− 2.128	56	− 0.102	(− 0.188, − 0.016)	0.020

The measurement data were normally distributed and the variances were assumed equal. Two independent samples t test(two-tailed) was used. In peripheral blood, the Bonferroni corrected *P* value was 0.05/32 (0.002).

#### MG severity and Bm1-Bm5 in peripheral blood

The Bm2 and Bm2 + Bm2 subsets were higher and the Bm3 + Bm4, eBm5, Bm5, and eBm5 + Bm5 subsets lower in the TMGH than in the T group. In addition, the Bm3 + Bm4 subset was lower in the TMGL than in the T group. The new index, (Bm2 + Bm2)/(eBm5 + Bm5), was significantly higher in the TMGH than in the T group, as well as being numerically but non-significantly higher in the TMGL than in the T group (Table 3).

**Table 3:** The expression levels of B cell subsets in peripheral blood in three group.

Subsets of B cells	T	TMGL	TMGH	Statistical test	Δmean (95%CI of Δmean) (T VS TMGL)	Δmean (95%CI of Δmean) (T VS TMGH)	Δmean (95%CI of Δmean) (TMGL VS TMGH)	*P* value
Bm1	10.0 ± 6.2	8.9 ± 5.7	12.3 ± 15.0	F = 0.584, df = 2	1.054 (− 5.298, 7.406)	− 2.351 (− 8.612, 3.910)	− 3.405 (− 9.910, 3.099)	0.569
Bm2	3.3 ± 1.5	4.3 ± 2.2	5.5 ± 2.6^a^	F = 5.136, df = 2	− 0.936 (− 2.278, 0.406)	− 2.178 (− 3.540, − 0.816)	− 1.243 (− 2.652, 0.167)	0.012
Bm2′	2.2 ± 0.4	2.1 ± 0.6	2.6 ± 0.8	F = 3.324, df = 2	0.101(− 0.297, 0.499)	− 0.415 (− 0.813, − 0.017)	− 0.516 (− 0.947, − 0.085)	0.062
Bm2 + Bm2′	5.2 ± 1.7	6.2 ± 2.7	7.6 ± 3.4	F = 3.850, df = 2	− 0.968 (− 2.684, 0.748)	− 2.404 (− 4.146, − 0.662)	− 1.436 (− 3.222, 0.350)	0.035
Bm3 + Bm4	21.0 ± 3.9	17.8 ± 4.0	17.7 ± 3.4	F = 4.821, df = 2	3.108 (0.723, 5.493)	3.216 (0.747, 5.685)	0.108 (− 2.520, 2.737)	0.011
eBm5	9.0 ± 2.8	8.6 ± 2.6	6.8 ± 2.7	F = 3.635, df = 2	0.435 (− 1.249, 2.118)	2.229 (0.518, 3.940)	1.794 (− 0.015, 3.603)	0.032
Bm5	9.6 ± 2.6	9.1 ± 2.9	7.6 ± 1.9	F = 3.503, df = 2	0.512 (− 1.047, 2.071)	2.028 (0.469, 3.586)	1.515 (− 0.165, 3.196)	0.036
eBm5 + Bm5	17.9 ± 4.6	17.7 ± 4.4	14.8 ± 3.2	F = 3.671, df = 2	0.286 (− 2.233, 2.805)	3.119 (0.677, 5.561)	2.833 (0.144, 5.522)	0.029
Ratio (Bm2 + Bm2′)/(eBm5 + Bm5)	0.3 ± 0.1	0.4 ± 0.2	0.5 ± 0.2^a^	F = 4.457, df = 2	− 0.044 (− 0.154, 0.066)	− 0.161 (− 0.271, − 0.051)	− 0.117 (− 0.232, − 0.002)	0.022

The measurement data were normally distributed. The one-way analysis of variance (ANOVA) was used. In peripheral blood, the Bonferroni corrected *P* value was 0.05/32 (0.002).

### Distributions of switched and non-switched memory B cells

#### Peripheral blood

CD19^+^CD27^+^ memory B cells were subdivided into two groups based on the expression of IgD. These cells were designated CD19^+^IgD^-^CD27^+^ switched memory B cells and CD19^+^IgD^+^CD27^+^ non-switched memory B cells. The ratio of CD19^+^IgD^-^CD27^+^ switched memory B cells to CD19^+^ B cells in the three groups (T, TMGL, TMGH) tended to decrease, with the differences being statistically significant. However, the ratio of CD19^+^IgD^+^CD27^+^ non-switched memory B cells to CD19^+^ B cells showed the same trend, indicating statistically significant differences. This ratio was significantly lower in the TMG than in the T group, indicating that the ratios of both non-switched memory B cells and switched memory B cells to CD19^+^ B cells were lower in the TMG group (Tables 4, 5).

**Table 4:** The expression levels of SMB and NSMB in peripheral blood in three group.

Subsets of B cells	T	TMGL	TMGH	Statistical test	Δmean (95%CI of Δmean) (T VS TMGL)	Δmean (95%CI of Δmean) (T VS TMGH)	Δmean (95%CI of Δmean) (TMGL VS TMGH)	*P* value
Switched memory B	0.3 ± 0.1	0.2 ± 0.1^a^	0.2 ± 0.1^a^	F = 5.396, df = 2	0.061 (0.010, 0.111)	0.078 (0.026, 0.131)	0.017 (− 0.040, 0.075)	0.007^a^
Non-switched memory B	0.1 (0.2–0.1)	0.1 (0.1–0.0)	0.1 (0.2–0.1)	H = 6.219, df = 2	0.092 (0.023, 0.161)	0.083 (0.015, 0.152)	− 0.009 (− 0.087, 0.069)	0.045^b^

^a^The measurement data were normally distributed and one-way analysis of variance (ANOVA) was used.

^b^The data did not follow a normal distribution and the Kruskal–Wallis test was used. In peripheral blood, the Bonferroni corrected *P* value was 0.05/32 (0.002).

**Table 5:** The expression levels of SMB and NSMB in peripheral blood in two group.

Subsets of B cells	T	TMG	Statistical test	Δmean	95%CI of Δmean	*P* value
Switched memory B	0.3 ± 0.1	0.2 ± 0.1	t = 3.119, df = 42.073	0.069	(0.024, 0.113)	0.003^a^
Non-switched memory B	0.1 (0.2–0.1)	0.1 (0.1–0.0)	–	–	–	0.014^b^

^a^The measurement data were normally distributed. Independent two-sample t-test (two-tailed) was used.

^b^The data did not follow a normal distribution. Mann–Whitney U test was used. The value of Mann–Whitney U test was 375.000 and Z score was − 2.470. In peripheral blood, the Bonferroni corrected *P* value was 0.05/32 (0.002).

#### The relationship between switched and non-switched memory B cells

The relationships between switched and non-switched memory B cells were evaluated in individual patients from the T, TMGL, TMGH, and TMG groups. The ratios of CD19^+^IgD^+^CD27^+^ non-switched memory B cells to CD19^+^ B cells were significantly lower than the ratios of CD19^+^IgD^-^CD27^+^ switched memory B cells to CD19^+^ B cells in the TMGL, TMGH, and TMG groups, but not in the T group. To clarify this relationship, the differences between the ratio of CD19^+^IgD^-^CD27^+^ switched memory B cells to CD19^+^ B cells and the ratio of CD19^+^IgD^+^CD27^+^ non-switched memory B cells to CD19^+^ B cells were calculated. The slope of the T group was significantly lower than that of the TMG and TMGL groups. Although the slope of the T group was also lower than that of the TMGH group, the difference was not statistically significant. Moreover, the slope of the TMGL group was greater larger than that of the TMGH group, although this difference was not statistically significant (Table 6) (Fig. S2).

**Table 6:** The expression levels of SMB and NSMB in peripheral blood in three group.

Group	Subsets of B cells		Statistical test	*P* value
	Non-switched memory B	Switched memory B		
T	0.1 (0.2–0.1)	0.3 ± 0.1	Mann–Whitney U = 357	0.037
TMGL	0.1 (0.1–0.0)	0.2 ± 0.1	Mann–Whitney U = 83	< 0.001
TMGH	0.1 (0.2–0.1)	0.2 ± 0.1	Mann–Whitney U = 132	0.026
TMG	0.1 (0.1–0.0)	0.2 ± 0.1	Mann–Whitney U = 480	< 0.001

The data did not meet the homogeneity of variance, and the Mann–Whitney test was use. In peripheral blood, the Bonferroni corrected *P* value was 0.05/32 (0.002).

#### Switched and non-switched memory B cells in the TME

To better comprehend the distribution of non-switched memory B cells, tumor tissue from 12 randomly selected patients in each group were subjected to multi-label immunofluorescence staining. The numbers of CD19^+^IgD^−^CD27^+^ switched and CD19^+^IgD^+^CD27^+^ non-switched memory B cells were quantified and their mean fluorescence intensities calculated. Fluorescence was significantly lower in the T group than in the TMG, TMGL, and TMGH groups, while being significantly higher in the TMGL than in the TMGH group. Fluorescence tended to be higher in the TMGH than in the T group, although this difference was not statistically significant (Table 7).

**Table 7:** Expression levels of SMB and NSMB in thymoma TME.

Group	Non-switched memory B						Switched memory B		
	MFI	Statistical test	Δmean (95%CI of Δmean) (T VS TMGL)	Δmean (95%CI of Δmean) (T VS TMGH)	Δmean (95%CI of Δmean) (TMGL VS TMGH)	*P* value	MFI	Statistical test	*P* value
T	84.9 ± 21.1	F = 11.40, df = 2	− 41.410 (− 66.580, − 16.240)	–	–	< 0.001^a^	33.9 (38.6–26.3)	H = 32.8, df = 2	< 0.001^b^
TMGL	126.3 ± 44.1		–	–	29.180 (4.330, 54.020)		59.2 (85.2–41.7)		
TMGH	97.1 ± 18.4		–	− 12.230 (− 27.070, 2.607)	–		32.0 (39.2–29.5)		
TMG	100.3 (126.4–87.6)	Mann–Whitney U = 225	–	–	–	< 0.001^c^	41.0 (60.3–32.0)	Mann–Whitney U = 235	< 0.001^c^

^a^The measurement data were normally distributed and one-way analysis of variance (ANOVA) was used.

^b^The data did not follow a normal distribution and the Kruskal–Wallis test was used.

^C^Compared to T group, Mann–Whitney U test was used. In the TME, the Bonferroni corrected *P* value was 0.05/4 (0.013).

**Figure 2:**
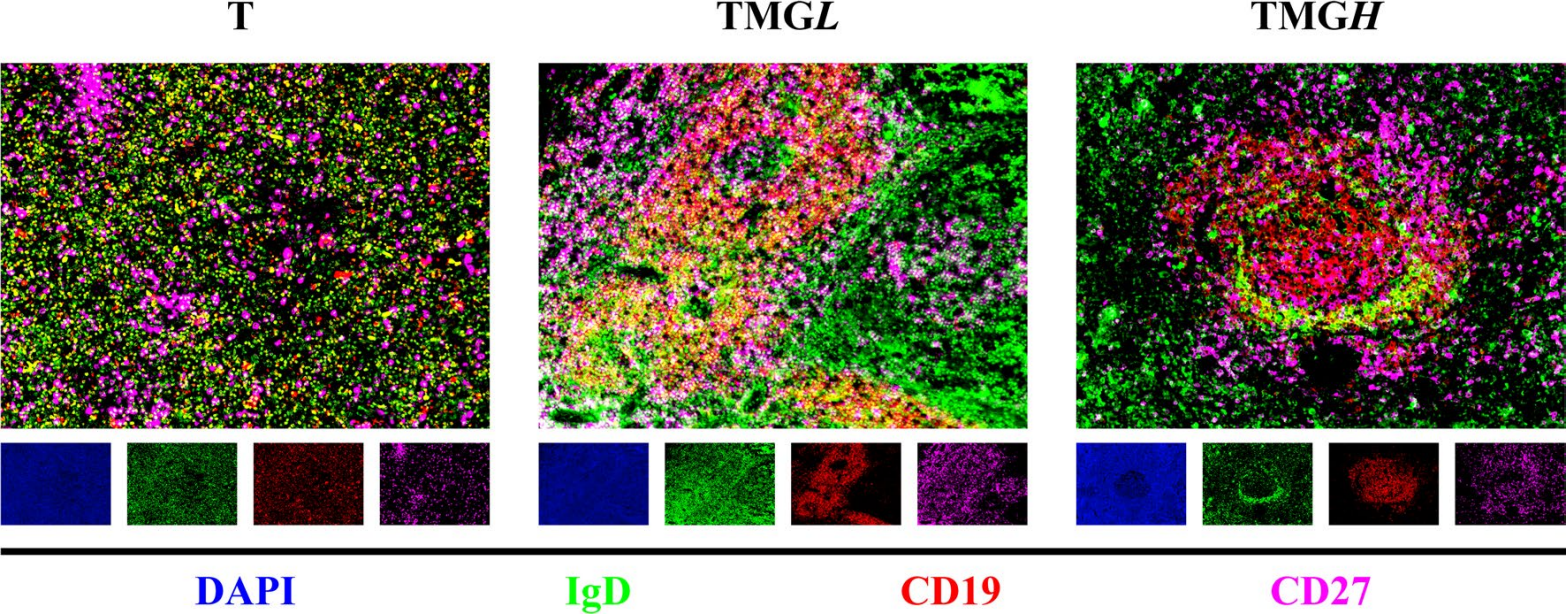
Switched memory B cells and non-switched memory B cells in TME. The expression of switched memory B cell and non-switched memory B cell in different groups. Multi-label immunofluorescence was used to label these two types of the cells. Multiplexed TSA was visualized using performing a triplex (CD19 in PPD 520, CD27 in PPD 570, IgD in PPD 650).

CD19^+^B cells aggregated to form tertiary lymphoid tissue in thymomas from patients in the TMGL and TMGH groups, whereas the distribution of CD19^+^B cells was diffuse in thymoma tissue from the T group. CD19^+^IgD^+^CD27^+^ non-switched memory B cells assembled around tertiary lymphoid tissue in thymomas, especially in patients with TMG, whereas a few scattered cells were observed in thymoma tissue from the T group (Fig. 2).

## Discussion

MG is the most common comorbidity of thymoma, with its pathogenesis being both similar to and different from pure MG. MG is a B cell-mediated autoimmune disease. To determine the pathogenesis of TMG, the present study evaluated the distribution of multi-polarized B cell subsets in the PB and TME of patients with TMG. Surface markers of immune cells are diverse and the immune microenvironment is a complex network. More B cell subsets need attention, especially in disease states such as TMG, in which tumors intersect with autoimmune diseases. The method of Bonferroni correction of P values in this study was relatively conservative, with the levels of critical P values corrected for large-scale multiple statistical tests to ensure true positives. Similarities between experimental results and corrected *P* values indicate that these experimental results are more statistically significant.

The frequency of CD19^+^ B cells in PB was found to be significantly higher in the TMG and TMGH groups than in the T group, with exacerbation of MG resulting in gradual increases in the percentages of peripheral CD19^+^ B cells. This trend was also observed in the thymoma TME. CD19^+^ B cells in the TME of pure thymoma were few and diffusely distributed, whereas CD19^+^ B cells in the TME of TMG patients were generally aggregated and participated in the formation of GCs, as well as being distributed in the PVS. Exacerbation of MG may increase the numbers of GCs in the TME. Type B thymomas were more likely be associated with autoimmune diseases such as MG than types A and AB thymomas. Type B thymomas tended to show an increasing severity of thymoma-associated MG, from type B1 to type B3.

The frequency of CD20^+^ B cells in the PB did not differ significantly in patient groups evaluated in the present study. The CD19/CD20 ratio was slightly higher in the TMG than in the T group, with this ratio showing a valley-like pattern, first decreasing and then increasing, in the TMGL and TMGH groups, although the differences were not statistically significant. This difference may be related to the increase in antibody-secreting cells in PB and the migration of early B cells to target organs in the TMGH group.

It has been shown that although both CD19 and CD20 expression are eventually lost from terminally differentiated plasma cells, CD19 expression remains on plasmoblasts and some plasma cells after CD20 expression is lost^22^. We wanted to evaluate the proportion of antibody-secreting cells in different patients by the level of CD19/CD20, as similar studies have been done in SLE^23^, in order to look for differences at the immune level. Although the results were not statistically significant, the higher percentage of CD19+ B cells in the TMG group than in the T group may be indicative of this, although of course a larger sample size would be needed to refine this part of the conclusion.

These results suggested that certain CD20^-^CD19^+^ B cells, such as long-lived plasma cells, play a critical role in the aggravation of MG^24^.

Switched memory B cells, which do not express IgD, undergo class switching and are indicators of normal B cell activation and development in lymph nodes and other secondary lymphoid tissue GCs^25^. The functional roles of B cell subsets in TMG were assessed by evaluating differences in CD19+ B cell subsets. Evaluation of Bm1-Bm5 cells showed that the ratios of Bm2, Bm2 + Bm2′, Bm3 + Bm4, and (Bm2 + Bm2′)/(eBm5 + Bm5) cells differed significantly in T, TMG, TMGL, and TMGH groups, regardless of whether two or three groups were compared. NSMBs and SMBs were in the middle or late stages of B cell development compared with naive B cells (Bm1), indicating that Bm2-Bm5 cells consisted at least in part of NSMBs and SMBs. Differences in B cell subsets after the Bm2 developmental stage were therefore clarified by measuring NSMBs and SMBs in PB and tissues.

The present study found that the trends of SMBs and NSMBs in thymoma TME and PB of patients in the T and TMG groups were opposite; i.e., SMBs and NSMBs in the TMG group were higher in tissue than in PB, whereas SMBs and NSMBs in the T group were higher in PB. This result confirmed the homing of B cells in response to thymomas. The GCs in the thymus responded to autoantigens and engendered AChR-specific plasma cells that were differentiated from manifold naive and antigen-specific Bmems. Moreover, the locations of GCs were associated with the expression and localization of autoantigens in the thymus. Small numbers of muscle-like myoid cells are present in the medulla of the thymus and express the fetal form of AChR, resulting in the generation of anti-AChR Abs and AChR-specific plasma cells in the thymus of these patients. The appearance of myoid cells confirmed that active GCs were generated in the thymus and that B cells differentiated and developed in the thymus to produce autoantibodies against AChR expressed by “myoid-like cells” during early stages of MG development^4,26^.

The present study showed that Bm2 and Bm2 + Bm2′ B cells, including those expressing CD19, tended to be higher in the PB of patients in the TMG than the T group, with Bm3 + Bm4, SMBs and NSMBs showing the opposite pattern. Exacerbation of MG increased the numbers of Bm2, Bm2 + Bm2′ and (Bm2 + Bm2′)/(eBm5 + Bm5) cells, while reducing the numbers of Bm3 + Bm4, eBm5, Bm5, and eBm5 + Bm5 cells, as well as SMBs and NSMBs. These findings suggested that patients cannot be evaluated based only on the frequencies of certain groups of CD19 and CD20 cells, and that various B cell subsets play an important role in the pathogenesis of TMG.

The Bm1–Bm5 classification of B cell subsets has been used to identify and compare various stages of circulating B cell differentiation in PB and tonsils^27^. These type-validated B cell subsets range from Bm1 (naive) to Bm5 (memory) B cells. One of the hallmarks of secondary immune responses is the recruitment of long-lived memory B cells secreting high-affinity antibodies specific for the triggering antigen. The B cell subpopulations derived from the GCs (Bm3 and Bm4) accumulated a large number of somatic mutations, whereas two of the mantle zone subpopulations (Bml and Bm2) displayed only IgM transcripts with virtually no evidence of having been subjected to somatic diversification. Interestingly, the remaining subset (Bm5), thought to represent the memory compartment, displayed the same level of somatic mutations seen in the GC subsets, further supporting their origin from GCs^27,28^.

The findings of this study suggest that early naive B cells and precursor B cells involved in GC formation were mobilized in patients with TMG and circulated in PB in large numbers. B cells in the GC may have been recruited back into the TME. Evaluation of patients with TMG should not only include a determination of the proportions of certain groups of Bm subgroup cells in the PB, but should be based on the (Bm2 + Bm2′)/(eBm5 + Bm5) ratios in these patients, allowing comprehensive determination of their condition.@@@

The present study also found that NSMBs and SMBs in PB had the same quantitative trend. NSMBs may develop from GCs independently, with most circulating NSMBs in PB deriving from the spleen^3,29^. NSMBs in tissues were found to increase with the aggravation of MG, indicating that NSMBs that need to undergo GC-development may only act in local tissues. Determination of IgH sequences of NSMBs with the same phenotype in tissues and PB could be used to clarify their origin. In contrast, this situation may be due to the aggravation of MG, which promoted the recruitment of more NSMBs and SMBs in the PB back to the thymoma TME. Because these patients have poor immune status, the increase of other B cell subsets, including plasma cells and Bm2 + Bm2′ cells, reduced the percentages of NSMBs and SMBs in the B cell subsets present in the PB.

Myasthenia gravis is associated with ectopic germinal centers in the thymus. Studies have shown increased expression of CXCL13 in the thymus and serum of glucocorticoid-untreated patients and decreased expression of CXCL13 after treatment, which is associated with improved clinical symptoms. Thymic extracts from glucocorticoid-untreated patients positively chemotactically attract normal B cells, and anti-CXCL13 antibodies inhibit this effect. In the thymus, epithelial cells preferentially produce CXCL13, whereas epithelial cells from MG patients overproduce CXCL13^30^. The MG thymus is a site of active neoangiogenic processes, including aberrant development of high endothelial venules (HEV). It was shown that HEV selectively expresses SDF-1 mRNA and presents SDF-1 protein on the luminal side, and that thymic HEV and associated chemokines mediate recruitment of peripheral cells to the MG thymus^31^. TLTs are present in both thymomas alone and thymomas associated with MG. The exacerbation of MG is associated with gradual increases in the numbers of GCs and the expression of CXCL13, proportional to changes in the numbers of CD19^+^ B cells, SMBs and NSMBs^1,32,33^.

There are some limitations to this study. Our study was a single-center cross-sectional study, so the number of cases was relatively limited. This explains why there was no statistical difference in the indicator CD19/CD20. For this reason, we expanded the patient review time to include more patients enrolled in the study while meeting the nadir criteria as much as possible, and the experimental data were reproducible. Reasonable data transformation can show the data differences more clearly. The histologic level was selected as much as possible for statistical analysis with multiple fields of view to fully assess the immune cell infiltration, and the experimental results have a certain degree of confidence. Due to the large number of B-cell subpopulation markers, other cell detection techniques can be introduced in the future to improve the definition of cells. Meanwhile, a multicenter joint study in the same region was conducted to further expand the sample size to improve this theory.

## Conclusion

**Figure 3:**
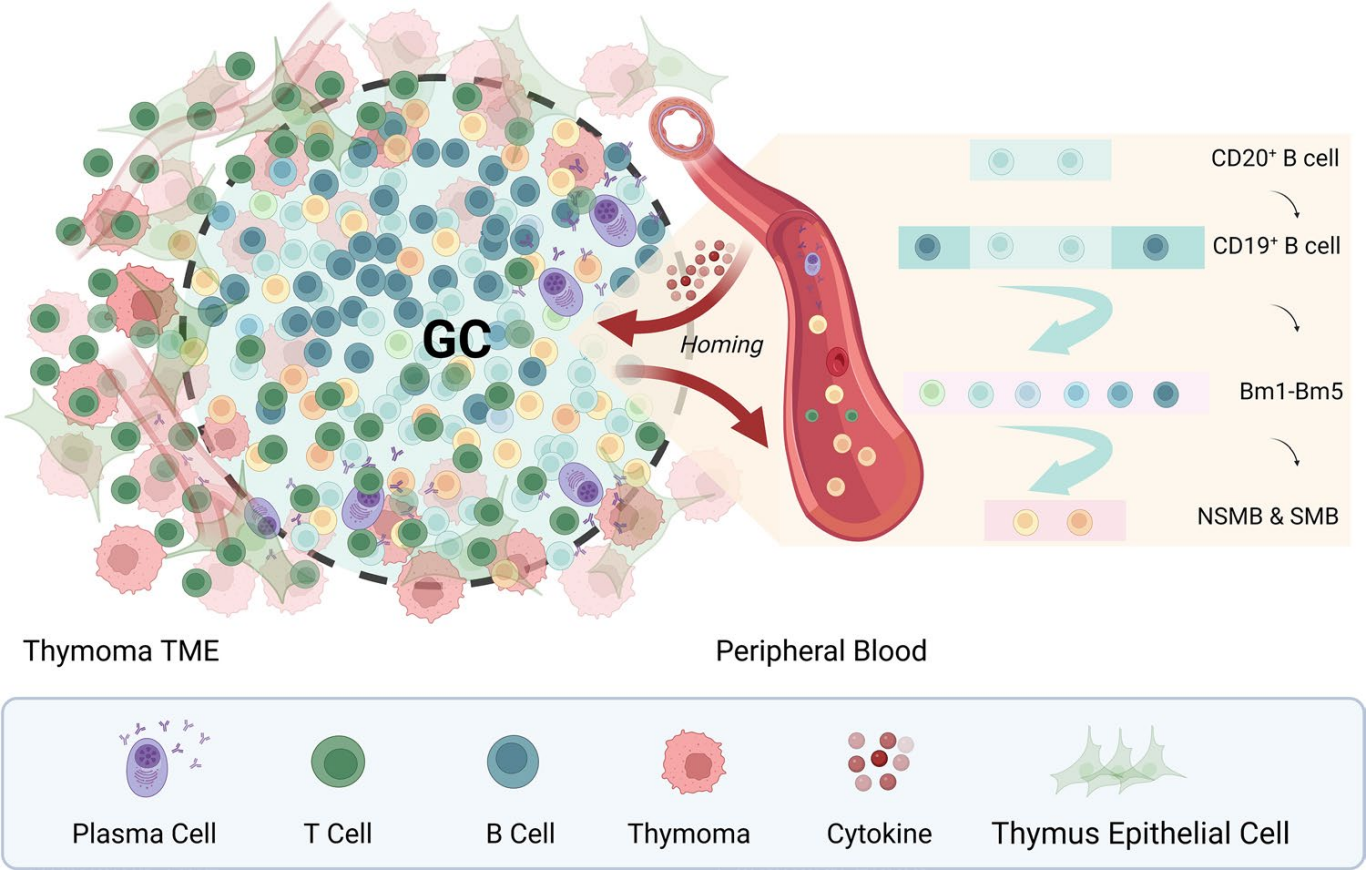
Links between tumor microenvironment and peripheral blood in thymoma-associated MG. Different B cell subsets existed in the peripheral blood of TMG patients, which may have a homing effect with thymoma TME under the action of cytokines. A germinal center-vascular-like structure existed in the thymoma TME, and memory B cells in peripheral blood were recruited to the TME, while a fraction of B cells that passed through the germinal center and under the helper activation of T cells differentiated into plasma cells for transport from the blood vessels to the periphery. The immune activity status of thymoma TME contributed to the generation of the immune inflammatory cascade of thymoma-associated MG from another perspective. Switched and non-switched memory B cells in peripheral blood had certain clinical guiding significance as new markers. Figure image created with BioRender.com.

The results of the present study suggest that the proportion of B cell subsets in PB is essential in thymoma patients with and without MG (Fig. 3).

B-cell subsets in patients with TMG may be used to evaluate the extent of MG and to predict the prognosis of TMG. As new markers, SMBs and NSMBs may be clinically useful in patients with severe TMG, as well as providing a new perspective for its treatment. Some Bmems may be recruited to the thymoma TME, as shown by the low frequency of SMBs in the peripheral blood of TMG patients and the high frequency of NSMBs in thymoma TME. Assembled NSMBs around GCs in the TME may undergo somatic hypermutation and class switching. Immune responses during the progression of MG from mild to severe show a fluctuating pattern in patients with TMG. Resection of the thymus is important for the treatment of TMG. Large-scale, multicenter cohort studies are needed to determine the time of peak immune response to improve the prognosis of TMG in Asians.

### Supplementary Information

Supplementary Figure S1.

Supplementary Figure S2.

Supplementary Table S1.

Supplementary Table S2.

Supplementary Table S3.

Supplementary Table S4.

Supplementary Table S5.

Supplementary Table S6.

## Data Availability

All data generated or analysed during this study are included in this published article.
