# Supplementary material for: Distribution of multi-level B cell subsets in thymoma and thymoma-associated myasthenia gravis
Source: Sci Rep. 2024 Feb 1;14:2674. doi: 10.1038/s41598-024-53250-6 (PMC10834956; doi:10.1038/s41598-024-53250-6)
Supplement: Supplementary file 1 — Supplementary Figure S1. [file 41598_2024_53250_MOESM1_ESM.docx]

**Distribution of multi-level B cell subsets in thymoma and thymoma-associated myasthenia gravis**

**Peng Zhang ^1#^**^*^**, Yuxin Liu ^1#^, Si Chen ^1^, Xinyu Zhang ^2^, Yuanguo Wang ^1^, Hui Zhang ^1^, Jian Li ^1^, Zhaoyu Yang ^1^, Kai Xiong ^1^, Shuning Duan ^1^, Zeyang Zhang ^1^, Yan Wang ^1^, Ping Wang ^3^, Huan Wang ^4^**

1 Department of Cardiovascular Thoracic Surgery, Tianjin Medical University General Hospital, Tianjin, China

2 School of Medicine, University of Dundee, UK

3 Tianjin Ruichuang Biological Technology Co. Ltd

4 Population and Precision Health Care, Ltd

* Correspondence: zhangpengtjgh@126.com; Tel.: +86 02260814720; Anshan Road No. 154, Heping District, 300052 Tianjin, China

# The two authors contribute equally.

**Supplementary Material**


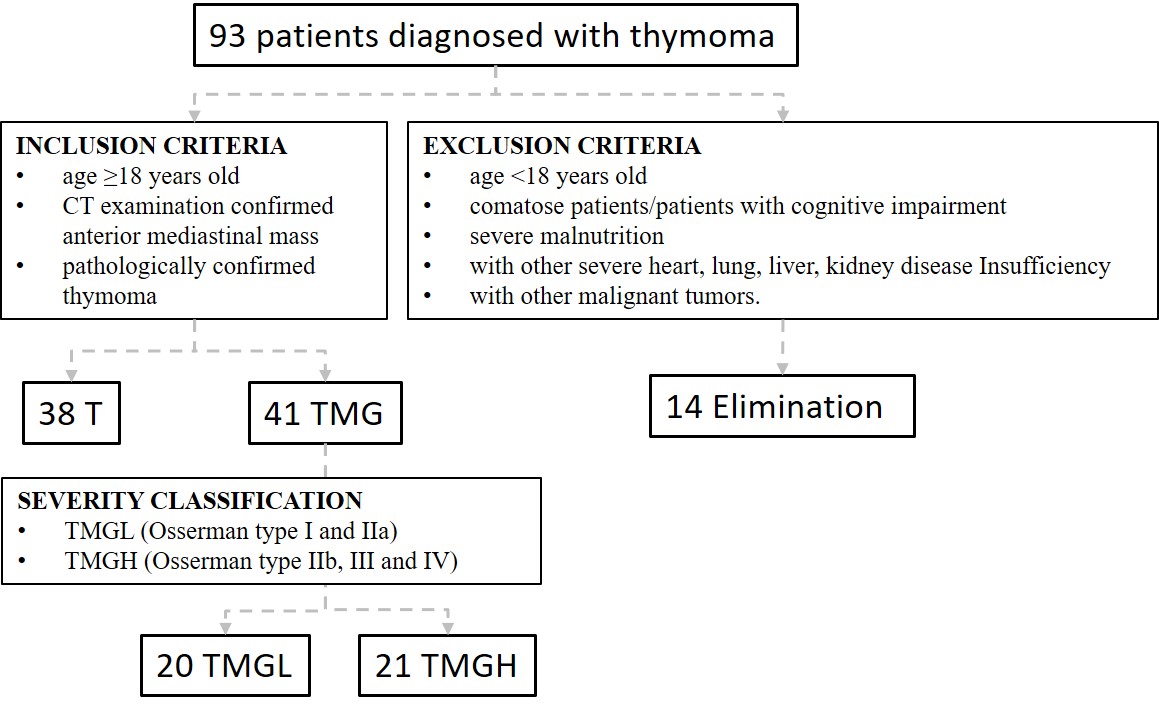


**Fig. S1.** Study Flowchart for Patient Recruitment.

93 patients diagnosed with thymoma who were admitted to Tianjin Meidical University General Hospital between February 2019 to March 2021. According to the inclusion and exclusion criteria, 14 cases were excluded.
