# Supplementary material for: Distribution of multi-level B cell subsets in thymoma and thymoma-associated myasthenia gravis
Source: Sci Rep. 2024 Feb 1;14:2674. doi: 10.1038/s41598-024-53250-6 (PMC10834956; doi:10.1038/s41598-024-53250-6)
Supplement: Supplementary file 2 — Supplementary Figure S2. [file 41598_2024_53250_MOESM2_ESM.docx]

**Distribution of multi-level B cell subsets in thymoma and thymoma-associated myasthenia gravis**

**Peng Zhang ^1#^**^*^**, Yuxin Liu ^1#^, Si Chen ^1^, Xinyu Zhang ^2^, Yuanguo Wang ^1^, Hui Zhang ^1^, Jian Li ^1^, Zhaoyu Yang ^1^, Kai Xiong ^1^, Shuning Duan ^1^, Zeyang Zhang ^1^, Yan Wang ^1^, Ping Wang ^3^, Huan Wang ^4^**

1 Department of Cardiovascular Thoracic Surgery, Tianjin Medical University General Hospital, Tianjin, China

2 School of Medicine, University of Dundee, UK

3 Tianjin Ruichuang Biological Technology Co. Ltd

4 Population and Precision Health Care, Ltd

* Correspondence: zhangpengtjgh@126.com; Tel.: +86 02260814720; Anshan Road No. 154, Heping District, 300052 Tianjin, China

# The two authors contribute equally.

**Supplementary Material**

**
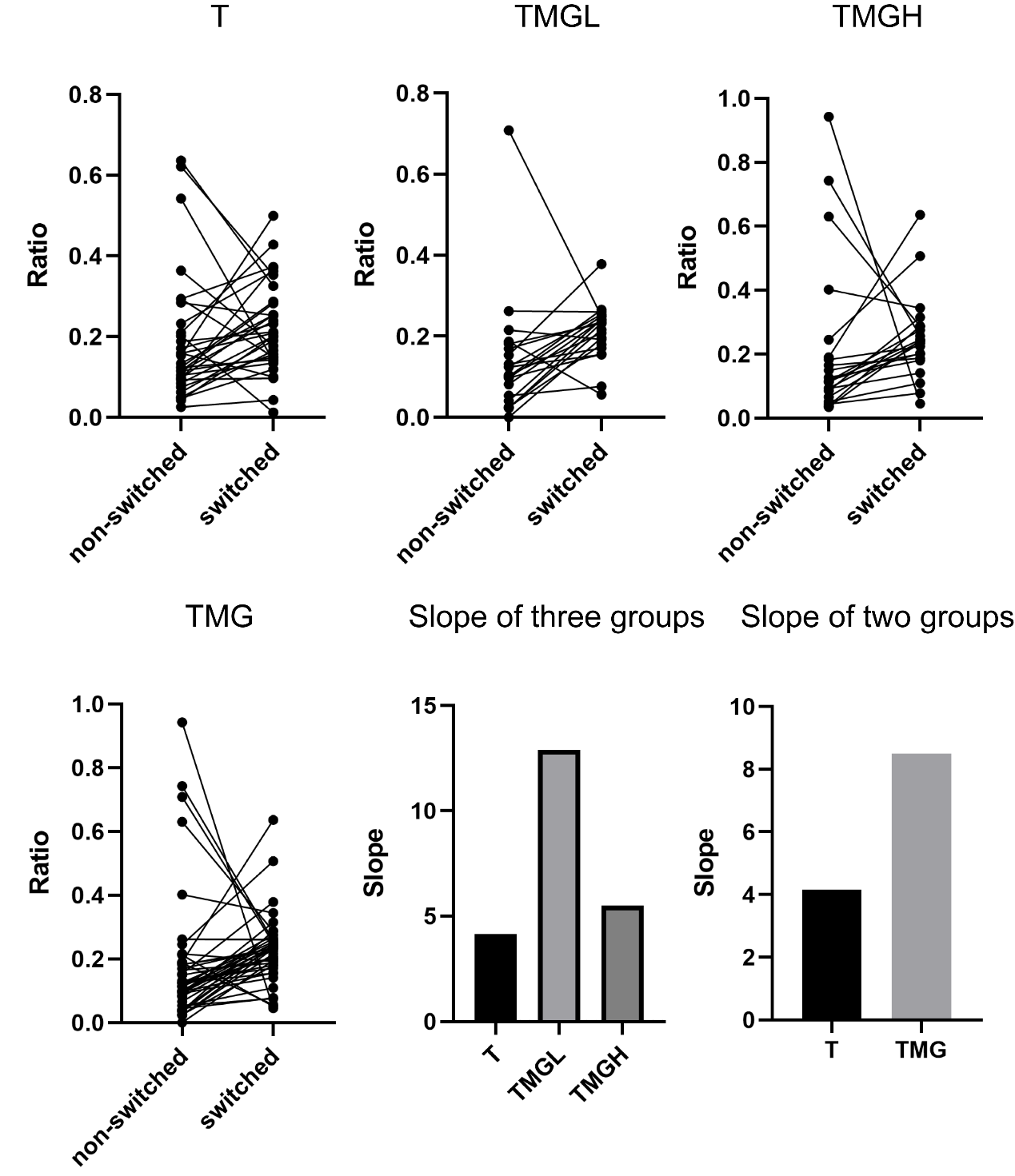
**

**Fig. S2.** The relationship between switched and non-switched memory B cell in different groups.

The ratios of switched memory B cell and non-switched memory B cell to CD19^+^B cell are investigated in the same patient to trace the relation. The slope (the difference between the ratio of switched memory B cell to CD19^+^ B cell and the ratio of non-switched memory B cell to CD19^+^ B cell) was compared in both three groups (T, TMGL, TMGH) and two group (T, TMG). Only the slope of T group did not obey the normal distribution. In the comparation of three groups, the Kruskal-Wallis test was used. *Kruskal-Wallis statistic*=5.725, *P*=0.057. In the comparation of two group, the Mann-Whitney test was used. *Mann-Whitney U*=404, *P*=0.023. The bar in each team means standard deviation of normally distributed data. The horizontal line represented the mean (normally distributed data) or median (non-normally distributed data).
