# Supplementary material for: Distribution of multi-level B cell subsets in thymoma and thymoma-associated myasthenia gravis
Source: Sci Rep. 2024 Feb 1;14:2674. doi: 10.1038/s41598-024-53250-6 (PMC10834956; doi:10.1038/s41598-024-53250-6)
Supplement: Supplementary file 3 — Supplementary Table S1. [file 41598_2024_53250_MOESM3_ESM.docx]

**Distribution of multi-level B cell subsets in thymoma and thymoma-associated myasthenia gravis**

**Peng Zhang ^1#^**^*^**, Yuxin Liu ^1#^, Si Chen ^1^, Xinyu Zhang ^2^, Yuanguo Wang ^1^, Hui Zhang ^1^, Jian Li ^1^, Zhaoyu Yang ^1^, Kai Xiong ^1^, Shuning Duan ^1^, Zeyang Zhang ^1^, Yan Wang ^1^, Ping Wang ^3^, Huan Wang ^4^**

1 Department of Cardiovascular Thoracic Surgery, Tianjin Medical University General Hospital, Tianjin, China

2 School of Medicine, University of Dundee, UK

3 Tianjin Ruichuang Biological Technology Co. Ltd

4 Population and Precision Health Care, Ltd

* Correspondence: zhangpengtjgh@126.com; Tel.: +86 02260814720; Anshan Road No. 154, Heping District, 300052 Tianjin, China

# The two authors contribute equally.

**Supplementary Material**

Table S1. The characteristics of the enrolled patients.

|  | T (n=38) | TMGL (n=20) | TMGH (n=21) | Statistical test | Δmean  (95%CI ofΔmean)  (T VS TMGL) | Δmean  (95%CI ofΔmean)  (T VS TMGH) | Δmean  (95%CI ofΔmean)  (TMGL VS TMGH) | *P Value* |
| --- | --- | --- | --- | --- | --- | --- | --- | --- |
| Gender |  |  |  |  |  |  |  |  |
| Female | 21 (46.7%) | 10 (22.2%) | 14 (31.1%) | χ²=1.247, df= 2 |  |  |  | 0.536 |
| Male | 17 (50.0%) | 10 (29.4%) | 7 (20.6%) |  |  |  |  |  |
| Age range | 25-74 | 31-74 | 21-71 |  |  |  |  | - |
|  |  | *mean ± SD* |  |  |  |  |  |  |
| Age | 54.5±14.2 | 53.4±13.2 | 51.7±14.4 | F=0.469, df= 2 | 1.097  (-6.601, 8.796) | 2.781  (-4.797, 10.358) | 1.683  (-7.024, 10.390) | 0.766 |
| BMI | 24.8±3.5 | 23.1±6.4 | 24.5±4.1 | F=0.997, df=2 | 1.758  (-7.542, 4.270) | 0.341  (-2.131, 2.814) | 1.416  (-1.424, 4.257) | 0.374 |
| Hospital Admission in days, HOD | 16.6±6.3 | 15.5±3.7 | 21.8±13.6 | F=3.513, df=2 | 1.182  (-3.457, 5.820) | -5.178  (-9.743, -0.613) | -6.360  (-11.605, -1.114) | 0.035 |
| Duration in ICU in days | 2.1±1.7 | 3.7±6.8 | 3.7±6.3 | F=1.156, df=2 | -1.647  (-4.306, 1.011) | -1.662  (-4.278, 0.955) | -0.014  (-3.021, 2.993) | 0.320 |
| Ventilator Service Time, VST | 3.4±3.2 | 3.1±2.6 | 47.5±194.3 | F=1.515, df=2 | 0.282  (-54.569, 55.132) | -44.099  (-98.087, 9.889) | -44.381  (-106.416, 17.654) | 0.226 |
| Hospitalization Costs, HC | 58842.7±19914.0 | 56322.0±13421.4 | 88993.3±57986.1 | F=6.15 , df=2 | 2520.598  (-16513.000, 21554.180) | -29440.000  (-47865.100, -11015.100) | -31961.000  (-53313.100, -10608.200) | 0.003 |
| Postoperative Drainage, PD | 460.7±452.3 | 474.2±369.1 | 320.2±270.6 | F=1.069, df=2 | -13.542  (-228.699, 201.615) | 140.420  (-71.355, 352.194) | 153.962  (-89.380, 397.303) | 0.348 |

One-way analysis of variance (ANOVA). Abbreviation: T = single thymoma; TMGL = thymoma associated with MG in less severe condition; TMGH = thymoma associated with MG in severe condition. The Bonferroni corrected P value was 0.05/29 (0.002). There was no statistically significant difference among the three groups in terms of gender, age, BMI, the duration of ICU (in days), ventilator service time (in hours), and postoperative drainage (in ml). In terms of hospital admission days and costs (in Chinese RMB), the TMGH group was higher than the remaining two groups, and the difference was statistically significant. Tumor volumes were calculated using the equation [ Π/6*( Length∗Width∗Height) ] and the maximum tumor dimension in three orthogonal planes (length, width, and height, in cm).
