# Supplementary material for: Distribution of multi-level B cell subsets in thymoma and thymoma-associated myasthenia gravis
Source: Sci Rep. 2024 Feb 1;14:2674. doi: 10.1038/s41598-024-53250-6 (PMC10834956; doi:10.1038/s41598-024-53250-6)
Supplement: Supplementary file 4 — Supplementary Table S2. [file 41598_2024_53250_MOESM4_ESM.docx]

**Distribution of multi-level B cell subsets in thymoma and thymoma-associated myasthenia gravis**

**Peng Zhang ^1#^**^*^**, Yuxin Liu ^1#^, Si Chen ^1^, Xinyu Zhang ^2^, Yuanguo Wang ^1^, Hui Zhang ^1^, Jian Li ^1^, Zhaoyu Yang ^1^, Kai Xiong ^1^, Shuning Duan ^1^, Zeyang Zhang ^1^, Yan Wang ^1^, Ping Wang ^3^, Huan Wang ^4^**

1 Department of Cardiovascular Thoracic Surgery, Tianjin Medical University General Hospital, Tianjin, China

2 School of Medicine, University of Dundee, UK

3 Tianjin Ruichuang Biological Technology Co. Ltd

4 Population and Precision Health Care, Ltd

* Correspondence: zhangpengtjgh@126.com; Tel.: +86 02260814720; Anshan Road No. 154, Heping District, 300052 Tianjin, China

# The two authors contribute equally.

**Supplementary Material**

**Table S2. The classification of the enrolled patients.**

|  | | T | TMGL | TMGH | Statistical test | *P Value* |
| --- | --- | --- | --- | --- | --- | --- |
| ***Classification*** | | | | |  |  |
| Thymoma pathologic classification: number of patients | A | 3 (3.8%) | 0 (0.0%) | 1 (1.3%) | Fisher’s Excat Test | 0.013^a^ |
|  | AB | 12 (15.2%) | 4 (5.1%) | 2 (2.5%) |  |  |
|  | B1 | 7 (8.9%) | 1 (1.3%) | 0 (0.0%) |  |  |
|  | B2 | 6 (7.6%) | 7 (8.9%) | 2 (2.5%) |  |  |
|  | B3 | 3 (3.8%) | 2 (2.5%) | 7 (8.9%) |  |  |
|  | mixed | 6 (7.6%) | 5 (6.3%) | 9 (11.4%) |  |  |
|  | unknown | 1 (1.3%) | 1 (1.3%) | 0 (0.0%) |  |  |
| Thymoma Masaoka-Koga stage: number of patients | Ⅰ | 15 (20.3%) | 6 (8.1%) | 6 (8.1%) | χ²=11.387, df=8 | 0.134 |
|  | Ⅱa | 6 (8.1%) | 7 (9.5%) | 3 (4.1%) |  |  |
|  | Ⅱb | 8 (10.8%) | 2 (2.7%) | 9 (12.2%) |  |  |
|  | Ⅲ | 5 (6.8%) | 5 (6.8%) | 1 (1.4%) |  |  |
|  | Ⅳa | 1 (1.4%) | 0 | 0 |  |  |
|  | Ⅳb | 0 | 0 | 0 |  |  |
| Tumor Volume | (*Median Q3- Q1*) | 26.7(77.0-9.4) | 16.8(23.6-4.7) | 8.6(15.7-3.9) | Chi-Square=4.884  df=2 | 0.087^b^ |
| Tumor Texture | Solid | 30 (40.0%) | 17 (22.7%) | 18 (24.0%) | χ²= 1.465,  df=2 | 0.481 |
|  | Solid & Cystic | 6 (8.0%) | 3 (4.0%) | 1 (1.3%) |  |  |
| MGFA Classification | Ⅰ | 0 | 15 (36.6%) | 3 (7.3%) | Fisher’s Exact Test | ＜0.001^c^ |
|  | Ⅱa | 0 | 4 (9.8%) | 6 (14.6%) |  |  |
|  | Ⅱb | 0 | 1 (2.4%) | 5 (12.2%) |  |  |
|  | Ⅲa | 0 | 0 | 6 (14.6%) |  |  |
|  | Ⅲb | 0 | 0 | 1 (2.4%) |  |  |
|  | Ⅳa | 0 | 0 | 0 |  |  |
|  | Ⅳb | 0 | 0 | 0 |  |  |

^a^ The difference between disease groups and pathological types was calculated by Fisher’s Exact Test. A weak correlation between disease group and pathological type was statistically significant, Cramer’s V=0.397, P=0.012. ^b^ Median and Q3-Q1were shown in the table. The difference between disease groups and tumor volume was calculated by Median Test. ^c^ The difference between disease groups and MGFA classification was calculated by Fisher’s Exact Test. A correlation between disease groups and MGFA classification was statistically significant, Cramer’s V=0.664, P＜0.001. The Bonferroni corrected P value was 0.05/5 (0.01).
