# Supplementary material for: Distribution of multi-level B cell subsets in thymoma and thymoma-associated myasthenia gravis
Source: Sci Rep. 2024 Feb 1;14:2674. doi: 10.1038/s41598-024-53250-6 (PMC10834956; doi:10.1038/s41598-024-53250-6)
Supplement: Supplementary file 5 — Supplementary Table S3. [file 41598_2024_53250_MOESM5_ESM.docx]

**Distribution of multi-level B cell subsets in thymoma and thymoma-associated myasthenia gravis**

**Peng Zhang ^1#^**^*^**, Yuxin Liu ^1#^, Si Chen ^1^, Xinyu Zhang ^2^, Yuanguo Wang ^1^, Hui Zhang ^1^, Jian Li ^1^, Zhaoyu Yang ^1^, Kai Xiong ^1^, Shuning Duan ^1^, Zeyang Zhang ^1^, Yan Wang ^1^, Ping Wang ^3^, Huan Wang ^4^**

1 Department of Cardiovascular Thoracic Surgery, Tianjin Medical University General Hospital, Tianjin, China

2 School of Medicine, University of Dundee, UK

3 Tianjin Ruichuang Biological Technology Co. Ltd

4 Population and Precision Health Care, Ltd

* Correspondence: zhangpengtjgh@126.com; Tel.: +86 02260814720; Anshan Road No. 154, Heping District, 300052 Tianjin, China

# The two authors contribute equally.

**Supplementary Material**

Table S3. Immune indicators of the enrolled patients.

|  | | **T**  **(n=38)** | | **TMGL**  **(n=20)** | | **TMGH**  **(n=21)** | Statistical test | *P Value* |
| --- | --- | --- | --- | --- | --- | --- | --- | --- |
| ***Autoimmune Situation*** | |  | | |  |  |  |  |
|  | *n* |  | |  | |  |  |  |
| AChR antibodies  number of patients | Positive at thymectomy | 11 (18.0%) | | 14 (23.0%) | | 12 (19.7%) | χ²=20.694, df= 2 | ＜0.001^a^ |
|  | Negative at thymectomy | 21 (34.4%) | | 0 | | 3 (4.9%) |  |  |
| Immune Indices | Anti-ANA Positive | 30 (41.7%) | | 13 (18.1%) | | 12 (16.7%) | χ²=0.911, df= 2 | 0.634^b^ |
|  | Other Abs Positive | 11 (15.3%) | | 4 (5.6%) | | 2 (2.8%) |  |  |
|  | *Mean ± SD* |  | |  | |  |  |  |
|  | C3 | 84.2±24.3 | | 81.7±27.9 | | 83.5±13.1 | F=0.081, df=2 | 0.922^c^ |
|  | C4 | 19.3±7.4 | | 20.0±8.4 | | 20.1±6.8 | F=0.122, df=2 | 0.885^d^ |
|  | *Median (Q3-Q1)* | |  |  | |  |  |  |
|  | IgG | 1100.0(1347.5-881.3) | | 1140.0(1375.0-942.8) | | 1210.0(1462.5-1022.5) | Chi-Square=1.529 | 0.466^e^ |
|  | IgA | 161.5(254.0-114.3) | | 208.5(314.5-125.0) | | 223.0(272.8-179.5) | Chi-Square=3.598 | 0.165^e^ |
|  | IgM | 101.8(155.8-69.9) | | 72.5(101.2-60.5) | | 95.0(149.3-69.6) | Chi-Square=3.578 | 0.167^e^ |
|  | CRP | 0.3(0.5-0.2) | | 0.2(0.4-0.1) | | 0.2(0.3-0.2) | Chi-Square=1.239 | 0.538^e^ |
|  | IgE | 20.1(58.6-10.2) | | 31.5(100.8-7.3) | | 64.1(208.5-18.0) | Chi-Square=5.385 | 0.068^e^ |
| Serum Protein Electrophoresis | ALB | 60.2(62.6-57.7) | | 61.3(63.7-57.7) | | 59.4(61.6-57.5) | Chi-Square=2.331 | 0.312 ^e^ |
|  | α1-globulin | 3.7(4.4-3.4) | | 3.6(3.9-3.2) | | 3.6(4.1-3.2) | Chi-Square=2.664 | 0.264 ^e^ |
|  | α2-globulin | 9.0(10.2-7.9) | | 8.6(9.1-8.2) | | 8.3(9.4-7.9) | Chi-Square=1.154 | 0.562 ^e^ |
|  | β1-globulin | 5.7(6.1-5.3) | | 5.5(6.0-5.3) | | 6.1(6.7-5.4) | Chi-Square=3.726 | 0.155 ^e^ |
|  | β2-globulin | 4.6(5.2-4.4) | | 4.4(5.0-4.0) | | 4.6(5.4-4.2) | Chi-Square=2.318 | 0.314 ^e^ |
|  | γ-globulin | 16.1(18.7-13.4) | | 15.4(18.1-14.3) | | 17.5(20.0-15.3) | Chi-Square=3.354 | 0.187 ^e^ |
|  | A/G | 1.5(1.7-1.3) | | 1.6(1.8-1.4) | | 1.5(1.6-1.4) | Chi-Square=2.774 | 0.250 ^e^ |

^a^ The difference between disease groups and the expression of anti-AChR Abs was calculated by Pearson Chi-Square Test. A correlation between disease groups and the expression of anti-AChR Abs was statistically significant, Cramer’s V=0.582, P<0.001. ^b^ The number of relevant patients was shown in the table. The difference between disease groups and the current item was calculated by Pearson Chi-Square Test. ^c^ The difference between disease groups and the quantity of C3 (or C4) was calculated by One-way ANOVA. In the C3 group, Δmean of (T VS TMGL) was 2.508 and the 95%CI ofΔmean was -10.100 to 15.115; Δmean of (T VS TMGH) was 0.453 and the 95%CI ofΔmean was -11.956 to 12.862; Δmean of (TMGL VS TMGH) was -2.055 and the 95%CI ofΔmean was -16.314 to 12.204. ^d^ In the C4 group, Δmean of (T VS TMGL) was -0.697 and the 95%CI ofΔmean was-4.812 to 3.418; Δmean of (T VS TMGH) was -0.928 and the 95%CI ofΔmean was -4.979 to 3.122; Δmean of (TMGL VS TMGH) was -0.231 and the 95%CI ofΔmean was -4.886 to 4.423. ^e^ The difference between disease groups and the current item was calculated by Independent-Samples Kruskal-Wallis Test.
