# Supplementary material for: Distribution of multi-level B cell subsets in thymoma and thymoma-associated myasthenia gravis
Source: Sci Rep. 2024 Feb 1;14:2674. doi: 10.1038/s41598-024-53250-6 (PMC10834956; doi:10.1038/s41598-024-53250-6)
Supplement: Supplementary file 6 — Supplementary Table S4. [file 41598_2024_53250_MOESM6_ESM.docx]

**Distribution of multi-level B cell subsets in thymoma and thymoma-associated myasthenia gravis**

**Peng Zhang ^1#^**^*^**, Yuxin Liu ^1#^, Si Chen ^1^, Xinyu Zhang ^2^, Yuanguo Wang ^1^, Hui Zhang ^1^, Jian Li ^1^, Zhaoyu Yang ^1^, Kai Xiong ^1^, Shuning Duan ^1^, Zeyang Zhang ^1^, Yan Wang ^1^, Ping Wang ^3^, Huan Wang ^4^**

1 Department of Cardiovascular Thoracic Surgery, Tianjin Medical University General Hospital, Tianjin, China

2 School of Medicine, University of Dundee, UK

3 Tianjin Ruichuang Biological Technology Co. Ltd

4 Population and Precision Health Care, Ltd

* Correspondence: zhangpengtjgh@126.com; Tel.: +86 02260814720; Anshan Road No. 154, Heping District, 300052 Tianjin, China

# The two authors contribute equally.

**Supplementary Material**

**Table S4. The expression levels of CD19 and CD20 in peripheral blood.**

|  | **T** | **TMG** | **t** | Δmean | 95%CI ofΔmean | *P Value* |
| --- | --- | --- | --- | --- | --- | --- |
| CD19 | 8.1±4.4 | 9.5±3.5 | -1.593^a^  df=71 | -1.470 | (-3.309, 0.370) | 0.116 |
| CD20 | 7.9±4.3 | 8.6±3.2 | -0.689^a^  df=77 | -0.786 | (-3.059, 1.486) | 0.493 |
| CD19/CD20 | 1.1(1.4-0.9) | 1.1 (1.3-1.0) | - | - | - | 0.439 ^b^ |

^a^ The measurement data were normally distributed and the variances were assumed equal and two independent samples t test was used. ^b^ The data did not follow a normal distribution and the Mann-Whitney U test was used. The standardized test statistic of independent-samples Mann-Whitney U Test was 0.774. In peripheral blood, the Bonferroni corrected P value was 0.05/32 (0.002).
