# Supplementary material for: Distribution of multi-level B cell subsets in thymoma and thymoma-associated myasthenia gravis
Source: Sci Rep. 2024 Feb 1;14:2674. doi: 10.1038/s41598-024-53250-6 (PMC10834956; doi:10.1038/s41598-024-53250-6)
Supplement: Supplementary file 7 — Supplementary Table S5. [file 41598_2024_53250_MOESM7_ESM.docx]

**Distribution of multi-level B cell subsets in thymoma and thymoma-associated myasthenia gravis**

**Peng Zhang ^1#^**^*^**, Yuxin Liu ^1#^, Si Chen ^1^, Xinyu Zhang ^2^, Yuanguo Wang ^1^, Hui Zhang ^1^, Jian Li ^1^, Zhaoyu Yang ^1^, Kai Xiong ^1^, Shuning Duan ^1^, Zeyang Zhang ^1^, Yan Wang ^1^, Ping Wang ^3^, Huan Wang ^4^**

1 Department of Cardiovascular Thoracic Surgery, Tianjin Medical University General Hospital, Tianjin, China

2 School of Medicine, University of Dundee, UK

3 Tianjin Ruichuang Biological Technology Co. Ltd

4 Population and Precision Health Care, Ltd

* Correspondence: zhangpengtjgh@126.com; Tel.: +86 02260814720; Anshan Road No. 154, Heping District, 300052 Tianjin, China

# The two authors contribute equally.

**Supplementary Material**

Table S5. Differences in pathological types of CD19 and CD20.

| Subject | Source | Type Ⅱ Sum of Squares | df | Mean Square | F | p value |
| --- | --- | --- | --- | --- | --- | --- |
| CD19 | Group*Type | 208.848 | 8 | 26.106 | 1.140 | 0.350 |
| CD20 | Group*Type | 217.71 | 8 | 27.214 | 0.991 | 0.452 |
| CD19/CD20 | Group*Type | 3.017 | 8 | 0.377 | 0.372 | 0.931 |

No interaction between MG severity and pathological type.
