# Supplementary material for: Distribution of multi-level B cell subsets in thymoma and thymoma-associated myasthenia gravis
Source: Sci Rep. 2024 Feb 1;14:2674. doi: 10.1038/s41598-024-53250-6 (PMC10834956; doi:10.1038/s41598-024-53250-6)
Supplement: Supplementary file 8 — Supplementary Table S6. [file 41598_2024_53250_MOESM8_ESM.docx]

**Distribution of multi-level B cell subsets in thymoma and thymoma-associated myasthenia gravis**

**Peng Zhang ^1#^**^*^**, Yuxin Liu ^1#^, Si Chen ^1^, Xinyu Zhang ^2^, Yuanguo Wang ^1^, Hui Zhang ^1^, Jian Li ^1^, Zhaoyu Yang ^1^, Kai Xiong ^1^, Shuning Duan ^1^, Zeyang Zhang ^1^, Yan Wang ^1^, Ping Wang ^3^, Huan Wang ^4^**

1 Department of Cardiovascular Thoracic Surgery, Tianjin Medical University General Hospital, Tianjin, China

2 School of Medicine, University of Dundee, UK

3 Tianjin Ruichuang Biological Technology Co. Ltd

4 Population and Precision Health Care, Ltd

* Correspondence: zhangpengtjgh@126.com; Tel.: +86 02260814720; Anshan Road No. 154, Heping District, 300052 Tianjin, China

# The two authors contribute equally.

**Supplementary Material**

Table S6. The interaction between MG severity and pathological type.

| Group | Type | CD19 | | CD20 | | CD19/CD20 | |
| --- | --- | --- | --- | --- | --- | --- | --- |
|  |  | Number | CD19 | Number | CD20 | Number | CD19/CD20 |
| T | A | 3 | 8.3±6.2 | 3 | 6.8±4.5 | 3 | 1.2±0.2 |
|  | AB | 12 | 6.7±3.7 | 12 | 8.2±3.8 | 12 | 1.0±0.5 |
|  | B1 | 7 | 8.0±5.5 | 7 | 7.1±3.2 | 7 | 1.1±0.6 |
|  | B2 | 6 | 7.7±6.8 | 6 | 10.8±3.5 | 6 | 0.6±0.5 |
|  | B3 | 3 | 6.1±5.3 | 3 | 3.2±2.2 | 3 | 2.0±2.2 |
|  | mixed | 6 | 6.3±5.4 | 6 | 10.9±13.0 | 6 | 1.8±2.5 |
| TMGL | AB | 4 | 9.8±1.3 | 4 | 9.0±1.9 | 4 | 1.1±0.2 |
|  | B1 | 1 | 2.3±0.0 | 1 | 10.0±0.0 | 1 | 0.2±0.0 |
|  | B2 | 7 | 7.4±5.2 | 7 | 9.4±4.5 | 7 | 0.9±0.4 |
|  | B3 | 2 | 7.8±1.0 | 2 | 9.9±4.0 | 2 | 0.8±0.2 |
|  | mixed | 5 | 10.5±3.4 | 5 | 7.2±1.8 | 5 | 1.5±0.4 |
| TMGH | A | 1 | 3.2±0.0 | 1 | 2.9±0.0 | 1 | 1.1±0.0 |
|  | AB | 2 | 13.2±0.5 | 2 | 10.8±1.0 | 2 | 1.2±0.2 |
|  | B2 | 2 | 10.3±2.6 | 2 | 10.4±1.5 | 2 | 1.0±0.1 |
|  | B3 | 7 | 14.5±7.1 | 7 | 11.7±6.0 | 7 | 1.3±0.3 |
|  | mixed | 9 | 10.8±2.2 | 9 | 8.0±4.0 | 9 | 1.8±1.2 |

The data description of the main effects of two factors (MG severity--group, pathological type--type) were shown.
